# Jordanian Men's Experience of Emotional Abuse in Marital Relationships: The Role of Marriage Length and Motivation

**DOI:** 10.3389/fpsyg.2021.689235

**Published:** 2021-07-27

**Authors:** Rula Odeh Alsawalqa, Yara Abdel Rahman Sa'deh, Maissa N. Alrawashdeh

**Affiliations:** ^1^Department of Sociology, The University of Jordan, Amman, Jordan; ^2^Department of Sociology and Anthropology, Doha Institute for Graduate Studies (DI), Doha, Qatar

**Keywords:** emotional abuse, male victims, intimate partner violence, marriage, female perpetrators

## Abstract

Though emotional abuse is one of the worst and most common types of intimate partner violence, it has not been investigated in Arabic literature. Thus, this study explored the prevalence of emotional abuse among married Jordanian men. Furthermore, the moderating roles of marriage length, marriage motivation, age, and area in the path to emotional abuse were investigated. An online survey was conducted using a random sample of Jordanian married men in Amman. A total of 1,003 participants with an average age of 42.51 and a marital relationship duration ranging from 1 to 53 years were selected. The results revealed that isolation was the most prevalent emotional abuse domain, followed by degradation, property damage, and sexual coercion. However, all emotional abuse domains were more prevalent among rural rather than urban men, in both traditional and love marriages. Emotional abuse was higher among men who married for love. Younger men reported experiencing higher emotional abuse levels, which declined with age and increasing marriage length. Further research is required to explore the nature of emotional abuse forms and their underlying reasons among married men, as differences in sociodemographic characteristics could affect the identification and understanding of emotional abuse and contribute to developing an intellectual framework capable of finding solutions for abusive marital relations in the Jordanian context.

## Introduction

Intimate partner violence (IPV) is mostly regarded as a gendered, heterosexual phenomenon that constitutes a significant social problem (Wright, 2016), and is framed within the context of human rights (Berthold, 2015). Violence in intimate relationships is referred to by various terms: wife abuse, wife battering, family violence, domestic violence, and gender-based abuse (Lien and Lorentzen, 2019). IPV is defined as any behavior within an intimate relationship that causes physical, psychological, or sexual harm to an individual (World Health Organization, 2002). Hence, it may express itself in the form of physical, psychological, emotional, verbal, or financial abuse (Australian Bureau of Statistics, 2016; Walby and Towers, 2018; Walker et al., 2020). Notably, many studies have found that men too fall victim to these forms of abuse by their female partners (Hines and Malley-Morrison, 2001; Stuart et al., 2006; Hogan, 2016; Perryman and Appleton, 2016; Huntley et al., 2019; Woodyard, 2019; Brooks et al., 2020; Joseph-Edwards and Wallace, 2020; Machado et al., 2020; Walker et al., 2020).

In society, men generally receive less recognition as IPV victims than women (Wright, 2016). Men may not report the emotional abuse perpetrated by women for the following reasons: a) the fact that violence toward men in intimate relationships is contrary to gender stereotypes (Steinmetz, 1977; Pagelow, 1983; Lien and Lorentzen, 2019) where men are socialized to tolerate and justify violence and to remain silent about it (Wright, 2016), b) the social stigma of being a male victim, and c) to protect their standards of masculinity (Migliaccio, 2001). Thus, society has treated this issue either with selective inattention or has dismissed it (Steinmetz, 1977), leading to the marginalization of male IPV victims (Wright, 2016).

The most prevalent and common form of IPV perpetrated by women is emotional abuse (Williams et al., 2008; Follingstad and Edmundson, 2010; Carney and Barner, 2012). Men experience the same emotional abuse that women experience, and there is much evidence that men experience emotional abuse by women partners (Hines and Malley-Morrison, 2001). Similarly, women are more likely to perpetrate emotional than physical aggression toward male partners (Hines and Saudino, 2003). Emotional abuse has neither gained adequate nor as much attention as other types of abuse, including physical and sexual abuse (Carney and Barner, 2012; Joshua, 2019; David, 2020), due to the prevalent notion that IPV is committed against women, either physically or sexually, rather than emotionally; few male victims realize that emotional abuse is a form of IPV (Hogan, 2016).

Moreover, emotional abuse is invisible, seldom recognized by victims, and difficult to describe, observe, or even discuss. Therefore, it is difficult to document it, and those who inflict this abuse or endure it are usually in varying states of denial (Barkhuizen and Pretorius, 2005; Smullens, 2007). This is because the abuser is initially enmeshed, or completely obsessed, with their partner. The abuser then becomes neglectful toward their partner and starts to make their partner feel invisible (Smullens, 2010), which is described as cycles of emotional abuse and includes enmeshment, extreme overprotection and overindulgence, complete neglect, rage, and rejection/abandonment (Smullens, 2007). Regarding neglect, Cohen (2013) argued that emotional neglect is distinct from emotional abuse. Emotional neglect involves acts of omission, that is, omitting behaviors that promote emotional well-being, whereas emotional abuse involves acts of commission, that is, committing acts against others that can be emotionally hurtful or traumatizing, such as name-calling and manipulation. This does not preclude the inclusion of emotional neglect as a form of emotional abuse used in escalating conflict among spouses, because emotional neglect implies the failure to provide support for partner's health, emotional development, nutrition, shelter, and safe living conditions, despite the partner's ability to do so, and failing to express positive feelings or showing no emotion in interactions with their partner (Ludwig and Rostain, 2009).

The concept of emotional abuse is open-textured, with no specific definition, but it can be understood within the context of intimate relationships as any non-physical behavior or attitude intended to control, subdue, punish, or isolate another person through humiliation or fear (Karakurt and Silver, 2013: 1). Other terms have been used synonymously with the term emotional abuse, namely psychological abuse, verbal abuse or aggression, symbolic abuse, non-physical abuse, and psychological acute victimization (Smullens, 2007; McHugh et al., 2013; Mouradian, 2020).

Notably, McHugh et al. (2013) discussed the ambiguities and complexities of defining psychological abuse. They concluded that labeling individuals as psychologically abusive is problematic due to the subjective nature of psychological abuse, implying that the definition should include the perpetrator's intent to harm and impact on the victim, similar to emotional abuse where the perpetrator's intent may not be clear. There has been a tendency to not focus on the intention of the perpetrator to harm the victim to avoid missing important aspects of the concept of psychological abuse and to consider the interaction context of abusive behavior and the subjective experience of the victim. Thus, psychological/emotional abuse includes many acts such as dominance, control, isolation, ridicule, threats of violence, insulting, and swearing.

Emotional abuse against men involves verbal, non-verbal, and non-physical forms of aggression, including the following acts: screaming, socially isolating, insulting, humiliating, demeaning, taking control of all the finances, terrorizing, threatening to hurt them, making them feel guilty, falsely accusing assault on them or their children, stalking, making them feel like failures or crazy, threatening to take custody of the children, withholding affection, stalking, diminishing one's sense of self-worth and/or dignity, and using manipulative behavior (Tracy, 2012a,b; McHugh et al., 2013; Mouradian, 2020). These behaviors can be considered interpersonal strategies that are utilized to create a subjective victim while maintaining power, control, and dominance (Zavala and Guadalupe-Diaz, 2018).

According to the National Domestic Violence Hotline Emotional Verbal Abuse (2021), a person may be in an emotionally abusive romantic or marital relationship if their partner attempts to exert control over them by committing the following acts: name-calling, insulting, or constantly criticizing them; acting jealous or possessive and not trusting them; isolating them from family, friends, or others; covertly monitoring their activities, including demanding to know where they go, who they contact, and how they spend their time; attempting to control what they wear, including clothes, makeup, or hairstyles; humiliating them in any way, especially in front of others; gaslighting them by (a) pretending not to understand or refusing to listen to them, (b) questioning their recollection of facts, events, or sources, (c) trivializing their needs or feelings, and (d) denying previous statements or promises; threatening their children and family members; damaging their belongings, including throwing objects, punching walls, kicking doors, and so forth; blaming them for their abusive behaviors; accusing them of cheating, or cheating themselves and blaming their partner's actions; cheating on them to intentionally hurt them and threatening to cheat again to suggest that they are “better” than them; and telling them that they are lucky to be with them or that they will never find someone better.

Stark (2010) suggested that there are gendered asymmetries in practices of “coercive control,” which include three aspects: emotional abuse, sexual coercion, and stalking or obsessive behavior (Carney and Barner, 2012). Emotional abuse is considered the main aspect and includes patterns of manipulation, control, surveillance, isolation, and intimidation (Zavala and Guadalupe-Diaz, 2018). It is a control tactic as powerful as physical abuse and is often a precursor to physical violence if the relationship deteriorates (Karakurt and Silver, 2013; Hannem et al., 2015). Thus, understanding emotional abuse based on distinct theories and prevention strategies, as a construct separate from physical violence, is required (Karakurt and Silver, 2013). Importantly, Zavala and Guadalupe-Diaz (2018) highlighted the significance of the interplay of emotional and physical abuse with dependency, attachment, and social problems, and that perpetrators denigrate victims psychologically, to the point where they are convinced that they have limited options outside of the abuse.

Mouradian (2020) described emotional abuse as the worst type of abuse in intimate relationships because of its role in establishing and maintaining the overall abusive dynamic of the intimate relationship. Marital emotional abuse has negative effects on the receiving partner. In this regard, Legg (2019) listed the main long- and short-term effects on the brain and body; short-term effects include feeling shame, hopelessness, fear, and confusion. More specifically, some short-term physiological effects of this abuse include moodiness, aches and pains, difficulty concentrating, and muscle tension. The long-term effects include insomnia, chronic pain, social withdrawal, loneliness, guilt, and anxiety.

Furthermore, emotional abuse may contribute to the development of chronic conditions, such as fibromyalgia and chronic fatigue syndrome. Although women are more likely to experience emotional abuse and more coercive elements of psychological aggression than men (Stark, 2007; Karakurt and Silver, 2013), there is a belief that men are more sensitive to emotional abuse than women and can tolerate physical abuse better (Tracy, 2012b). Thus, there is a tendency that men's risk of emotional abuse increases while women's risk may be decreasing. The factors contributing to this shift could be because women have support to access more resources and the strengthened legislation (Karakurt and Silver, 2013). Regarding potential gender differences, a study reported that the frequency of emotional abuse did not differ between men and women, and the frequency of emotional abuse was positively associated with symptoms of depression and PTSD in both men and women (Bushong, 2018).

The prevalence and incidence of IPV perpetrated by women against men have steadily gained attention from Western and European researchers who have investigated the emotional abuse experiences of men in heterosexual relationships (Zavala and Guadalupe-Diaz, 2018). IPV against men in Arab countries has not yet been comprehensively studied, specifically emotional abuse, with this phenomenon having been largely neglected in social science and psychological research. Also, IPV victimization among males is mentioned in very few electronic newspaper articles, and the relevant prevalence and incidence figures are presented as marginal numbers in national surveys and statistical reports.

To our knowledge, this is the first study to address emotional abuse among men in marital relationships in Jordan. In a qualitative study, Alsawalqa (2021) found that Jordanian married men experienced psychological, emotional, and verbal abuse, coercive control, emotional neglect, and physical violence from their wives, and abusive wives used sex, children, isolation, and money as tactics to enable abuse. The major causes of abuse against the Jordanian husbands were wives' neglecting the children, house, appearance, and personal hygiene; wasting money; the wives' family interfering in the couple's private marital affairs; and the wife's betrayal.

Some Jordanian married men confirmed that the clan thinking and stereotype of women as the fairer sex, physically weak, and always the victim allowed them to abuse men and prevented them from reporting abuse because they would not be believed. They are also exposed to social stigma, insults, and humiliation if abused by women, particularly with physical abuse. Additionally, they feel useless, impotent, weak, and in despair due to the emotional neglect and psychological abuse from their wives (Alsawalqa, 2021).

Cultural norms and gender roles differentially influence men and women in their emotional expressions. Stereotypically, men are required to repress their emotions, hence, they are unable to express some of their feelings, including intimate feelings (Kelly and Hutson-Comeaux, 1999; Wester et al., 2002). According to Arab cultural norms, men crying to express their sadness is considered shameful and a diminution of their masculinity. The term “Man-box” was coined in an attempt to focus on some of the cultural and social barriers that cause men to suppress their emotions and impose on them expectations of aggressive and/or dominant behavior in social settings. This curtails men's freedom of emotional expression (Heilman et al., 2017), including choosing a life partner. A traditional marriage is imposed on most of them, where the wife is chosen by the family members of the man, such as the parents, or a professional matchmaker, without taking into account the importance of acceptance and feelings of love between prospective spouses. Other criteria are focused upon instead such as a man's ability to sustain his wife and provide housing, and the employment status, age, and lineage of the wife. The tribe/clan to which the family of each of the spouse belongs to is often a prerequisite for this type of marriage. Traditional marriages are still common in rural and urban areas of Jordan. As such, the goal of this study was not only to explore married men's experiences of emotional abuse by their wives, but also to examine how emotional abuse is correlated with age, area (rural, urban), marriage length, and marriage motivation (traditional and love marriage). Notably, there is limited literature on the role of these fundamental factors in emotional abuse. Previous studies have focused on emotional abuse tactics and prevention strategies, without examining motivation and demographic factors. Marriage length, and marriage motivation are two of the factors which have not been focused on in previous studies. This study therefore advances the literature on domestic abuse.

## Materials and Methods

### Participants and Data Collection

This study included 1,003 married men who were randomly selected from Amman city (including rural and urban areas), the capital of the Hashemite Kingdom of Jordan. On average, the male population in urban areas (80.3%, *n* = 805) was higher than that in rural areas (19.7%, *n* = 198).

The mean duration of marital relationships was 16.09 years, ranging from 1 to 53 years. In total, 36.7% (*n* = 368) of the participants reported being in a love marriage, and 63.3% (*n* = 635) reported being in a traditional marriage. Participants' mean age was 42.51 years (SD = 8.850), ranging from 19 to 74 years. Data were collected through a survey distributed to married men. The survey was conducted through an online anonymous survey platform using Google Forms. It was distributed via Facebook and WhatsApp, and could be accessed through an anonymous link. Each participant was advised that their participation was voluntary and there would be no consequences if they chose to withdraw from the survey. All participants provided informed consent when they agreed to fill out the questionnaire. No separate informed consent form was signed. This was done to preserve their privacy due to the sensitivity of the topic of study. In designing the survey, we took into consideration the Pew Research Center's (2021) instructions and recommendations for electronic surveys, which assisted us in offsetting the significant biases resulting from undercoverage and non-response, and in enabling the respondents to accurately complete and return the survey. Ethical approval for this study was granted by the Institutional Review Board (IRB) of the University of Jordan, the Hashemite Kingdom of Jordan (reference number IRB/19/2021/86). Table 1 presents the demographic characteristics of the participants.

**Table 1:** Participants' demographic characteristics (*N* = 1,003).

**Demographics**	***N***	**%**	**Mean**	***SD***
Age			42.51	8.850
Area				
Urban	805	80.3		
Rural	198	19.7		
Marriage length			16.09	10.133
Marriage motivation				
Traditional marriage	635	63.3		
Love marriage	368	36.7		

### Measures

#### The Emotional Abuse Questionnaire

The Emotional Abuse Questionnaire developed by Waltz et al. (1994) was used in this study. It consists of 66 items assessing the level of emotional abuse that one experiences within their marital relationship, classified under four sub-scales: *Isolation* (24 items) including “My partner disapproves of my friends” and “My partner keeps me from going to places I want to go”; *Sexual Abuse* (7 items) including “My partner is not sensitive to me during sex” and “I feel pressured to have sex when I don't want to”; *Degradation* (28 items) including “My partner insults my family” and “My partner questions my sanity”; and *Property Damage* (7 items) including “My partner intentionally damages things I care about” and “My partner threatens to destroy my property.” Each item is rated on a 4-point Likert scale as follows: 1 = *Never*, 2 = *Rarely*, 3 = *Occasionally*, and 4 = *Very Often*. This questionnaire has been widely used in previous research on male victims of female-perpetrated partner violence.

### Data Analysis

**Figure 1:**
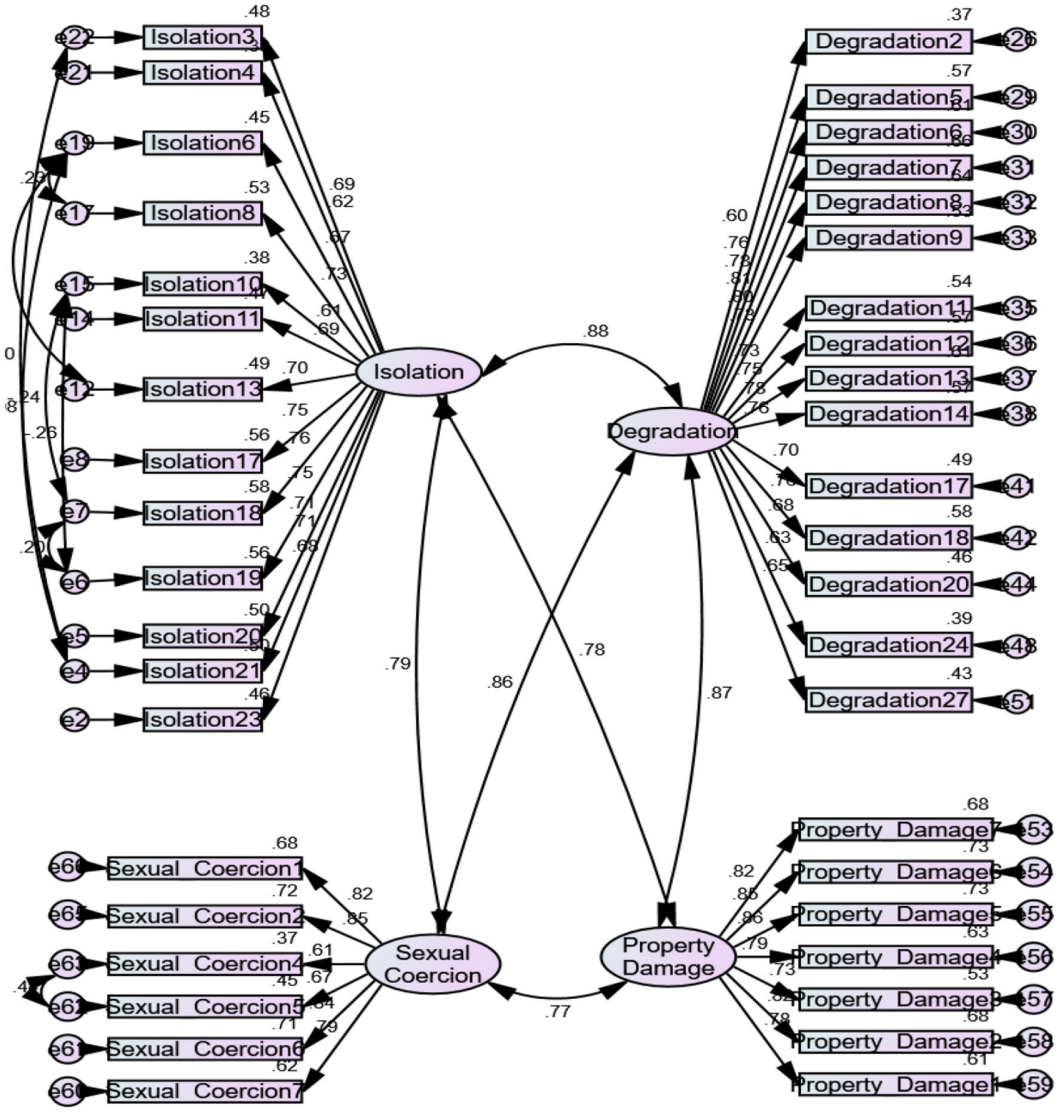
Confirmatory factor analysis.

Statistical analyses were performed using IBM SPSS-25. Cronbach's alpha coefficients were calculated to assess the reliability of our adjusted Emotional Abuse Questionnaire, using composite reliability. The coefficient alphas for the initial scale, including Isolation, Degradation, Sexual Abuse, and Property Damage subscales, were 0.933, 0.955, 0.854, and 0.930, respectively. The frequency distribution and descriptive statistics were calculated. Confirmatory factor analysis was run using IBM AMOS-24. The results showed that some items had low factor loadings (< 0.60) with their respective constructs, and thus were removed. The final measurement model, as shown in Figure 1, indicates acceptable goodness-of-fit measures, including RMSEA (0.061), SRMR (0.051), CFI (0.903), and chi-square/Df (4.781). Finally, convergent and discriminant validity were assessed.

## Results

### Summary Statistics

As shown in Table 2, descriptive statistics were calculated, including the means and standard deviations of all the domains of emotional abuse. Isolation was the most prevalent, followed by degradation, property damage, and sexual coercion.

**Table 2:** Descriptive statistics of the emotional abuse domains.

**Domains**	**Mean**	**Std. deviation**
Isolation	1.9640	0.59684
Degradation	1.9428	0.64045
Sexual abuse	1.6190	0.65435
Property damage	1.6614	0.65435
Total emotional abuse	1.7968	0.59966

Tables 3, 4 present the prevalence rate of each domain of emotional abuse in rural and urban areas of Amman. The results showed that the experience of all emotional abuse domains was more prevalent in rural rather than urban people.

**Table 3:** Means and Std. deviation of emotional abuse victimization by area.

**Subscales**	**Area**	***N***	**Mean**	**Std. deviation**
Isolation	Rural	198	2.1420	0.58526
	Urban	805	1.9202	0.59185
Degradation	Rural	198	2.0581	0.68211
	Urban	805	1.9145	0.62699
Sexual coercion	Rural	198	1.9185	0.72347
	Urban	805	1.5453	0.61470
Property damage	Rural	198	1.8752	0.80297
	Urban	805	1.6089	0.75006

**Table 4:** Analysis of variance for emotional abuse domains between rural and urban areas.

**Variables**	**Sum of squares**	**df**	**Mean square**	***F***	**Sig**.	
Isolation	Between groups	7.818	1	7.818	22.417	0.000
	Within groups	349.113	1,001	0.349		
	Total	356.931	1,002			
Degradation	Between groups	3.278	1	3.278	8.047	0.005
	Within groups	407.720	1,001	0.407		
	Total	410.998	1,002			
Sexual coercion	Between groups	22.125	1	22.125	54.427	0.000
	Within groups	406.912	1,001	0.407		
	Total	429.036	1002			
Property damage	Between groups	11.270	1	11.270	19.473	0.000
	Within groups	579.338	1,001	0.579		
	Total	590.608	1,002			

### Correlation Analysis

To assess the size and direction of the linear relationship of emotional abuse with age, area (rural or urban), marriage length, and marriage motivation, a bivariate Pearson's product-moment correlation coefficient (*r*) was calculated. The results of the correlation analysis are shown in Table 5. All relationships, except the relationship between marriage motivation and area, were statistically significant at *p* < 0.01. Emotional abuse was negatively correlated with age (*r* = −0.117, *p* < 0.01), area (*r* = −0.167, *p* < 0.01), and marriage length (*r* = −0.159, *p* < 0.01). Moreover, emotional abuse was positively correlated with marriage motivation (*r* = 0.119, *p* < 0.01). The negative association between emotional abuse and age indicates that emotional abuse decreased with increasing age. The negative association between emotional abuse and area suggests that emotional abuse was lower in urban areas. The negative association between emotional abuse and marriage length indicates that emotional abuse reduced with increasing marriage length. Finally, the positive association between emotional abuse and marriage motivation suggests that emotional abuse was higher in people who married out of love.

**Table 5:** Correlation analysis.

**Pearson correlation**		**Age**	**Area**	**Marriage length**	**Marriage motivation**	**Emotional abuse**
Age	Pearson correlation	1	0.096^**^	0.855^**^	−0.242^**^	−0.117^**^
	Sig. (2−tailed)		0.002	0.000	0.000	0.000
	N	1,003	1,003	1,003	1,003	1,003
Area	Pearson correlation	0.096^**^	1	0.111^**^	−0.043	−0.167^**^
	Sig. (2−tailed)	0.002		0.000	0.169	0.000
	*N*	1,003	1,003	1,003	1,003	1,003
Marriage length	Pearson correlation	0.855^**^	0.111^**^	1	−0.269^**^	−0.159^**^
	Sig. (2−tailed)	0.000	0.000		0.000	0.000
	*N*	1,003	1,003	1,003	1,003	1,003
Marriage motivation	Pearson correlation	−0.242^**^	−0.043	−0.269^**^	1	0.119^**^
	Sig. (2−tailed)	0.000	0.169	0.000		0.000
	*N*	1,003	1,003	1,003	1,003	1,003
Emotional abuse	Pearson correlation	−0.117^**^	−0.167^**^	−0.159^**^	0.119^**^	1
	Sig. (2−tailed)	0.000	0.000	0.000	0.000	
	*N*	1,003	1,003	1,003	1,003	1,003

** *Correlation is significant at the 0.01 level (2-tailed)*.

### Analysis of Variance

A two-way between-group analysis of variance (ANOVA) was conducted to explore the impact of area and marriage motivation on emotional abuse. Participants were divided into two groups according to area (group 1: rural; group 2: urban) and marriage motivation (group 1: traditional marriage; group 2: love marriage). Mean values, as illustrated in Table 6, showed that overall emotional abuse was higher in love marriages (*M* = 1.8904, *SD* = 0.58900) than in traditional marriages (*M* = 1.7426, *SD* = 0.59955). The results also showed that rural individuals experienced higher emotional abuse than their urban counterparts regardless of whether they were in a traditional or a love marriage.

**Table 6:** ANOVA: descriptive statistics.

**Area**	**Marriage motivation**	**Mean**	**Std. deviation**	***N***
**Dependent variable: emotional abuse**				
Rural	Traditional marriage	1.9417	0.64166	117
	Love marriage	2.0804	0.63031	81
	Total	1.9984	0.63910	198
Urban	Traditional marriage	1.6976	0.58088	518
	Love marriage	1.8368	0.56652	287
	Total	1.7472	0.57930	805
Total	Traditional marriage	1.7426	0.59955	635
	Love marriage	1.8904	0.58900	368
	Total	1.7968	0.59966	1003

As illustrated in Table 7, the interaction effect between area and marriage motivation was not statistically significant [*F*_(1, 999)_ = 0.000, *p* = 0.995]. There was a statistically significant main effect for area [*F*_(1, 999)_ = 26.118, *p* = 0.000] and marriage motivation [*F*_(1, 999)_ = 8.480, *p* = 0.004]. These results suggest that the scores of the emotional abuse domains were significantly different between urban and rural areas, as well as between traditional and love marriage.

**Table 7:** Tests of between-subjects effects.

**Source**	**Type III sum of squares**	**df**	**Mean square**	***F***	**Sig**.
**Dependent variable: emotional abuse**					
Corrected model	14.530^a^	3	4.843	13.993	0.000
Intercept	2170.516	1	2170.516	6270.898	0.000
Area	9.040	1	9.040	26.118	0.000
Marriage motivation	2.935	1	2.935	8.480	0.004
Area * marriage motivation	1.538E-5	1	1.538E-5	0.000	0.995
Error	345.779	999	0.346		
Total	3598.560	1003			
Corrected total	360.309	1002			

a *R Squared = 0.040 (Adjusted R Squared = 0.037)*.

### Reliability and Validity

Cronbach's alpha and composite reliability (CR) values >0.70 were used to evaluate the reliability of each latent variable (Hair et al., 2019). The Cronbach's alpha and CR values of all latent variables were >0.70, suggesting construct reliability. Factor loading values >0.60 and average variance extracted (AVE) values >0.50 were then used to determine convergent validity (Hair et al., 2019). All factor loading values were >0.60, which is considered appropriate. Furthermore, an AVE value of 0.50 or higher suggests that the latent construct, on average, accounts for 50% or more of the variance in the observed variables. A measure of discriminant validity is the heterotrait–monotrait ratio of correlations (HTMT), which measures the true correlations between constructs (Hair et al., 2019). Discriminant validity is present when HTMT values are <0.90. All values were less than the recommended value of 0.90, as evident in Table 8 and Figure 1; thus, discriminant validity was ensured.

**Table 8:** Discriminant validity (HTMT analysis).

**Latent variables**	**Isolation**	**Degradation**	**Property damage**
Degradation	0.872		
Property damage	0.764	0.872	
Sexual coercion	0.806	0.872	0.794

### Multi-Group Modeling

Multi-group modeling was used to compare the effects of age and marriage length on the domains of emotional abuse across the two marriage motivations: traditional marriage (*n* = 635) and love marriage (*n* = 368). Two models were investigated for the multi-group study: unconstrained and completely constrained (for traditional and love marriage). The fully constrained model demands that all estimated parameters be equal across classes, whereas the unconstrained model allows for variations in the estimated parameters (Byrne, 2013). Compared to the fully constrained model (χ^2^ = 8758.038, df = 1735), the unconstrained model better fitted the data (χ^2^ = 8655.794, df = 1690). The chi-square difference between these two models was also significant [Δχ^2^ = 102.244; Δdf =4 5; *p* (d) = 0.001], suggesting that the two groups (traditional and love marriage) were not equal.

**Figure 2:**
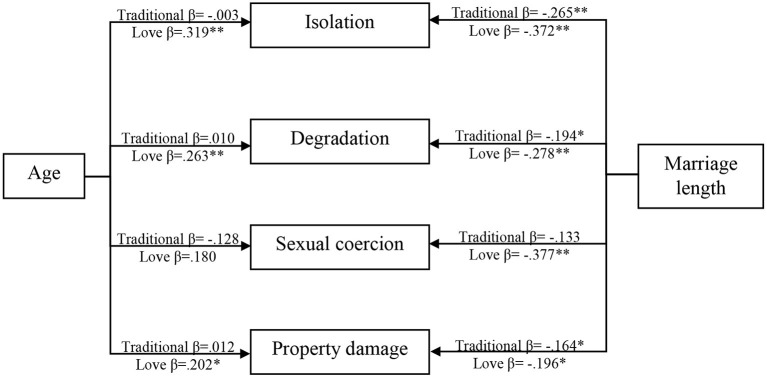
Multi-group modeling between traditional and love marriages.

The results in Table 9 and Figure 2 show that marriage length had a significant negative effect on isolation, degradation, and property damage in traditional marriages, indicating that emotional abuse decreases with marriage length. However, the other paths were not significant in the traditional marriage group. In the love marriage group, all the paths were significant except for the path from age to sexual coercion. Also, age had a significant positive impact on isolation, degradation, and property damage, indicating that when age increases, emotional abuse in terms of isolation, degradation, and property damage also increases in love marriages. Finally, marriage length had a significant negative effect on sexual coercion, isolation, degradation, and property damage in love marriages, suggesting that emotional abuse decreases with marriage length.

**Table 9:** Multi-group modeling: interaction of marriage motivation on associations of age and marriage length with emotional abuse victimization.

**Path relationship**			**Traditional marriage**			**Love marriage**		
			**Beta**	***T***	***P***	**Beta**	***T***	***P***
Isolation	< – – –	Age	−0.003	−0.041	0.967	0.319^**^	3.199	0.001
Degradation	< – – –	Age	0.010	0.136	0.892	0.263^**^	2.650	0.008
Sexual coercion	< – – –	Age	−0.128	−1.660	0.097	0.180	1.851	0.064
Property damage	< – – –	Age	0.012	0.159	0.873	0.202^*^	2.061	0.039
Isolation	< – – –	Marriage length	−0.265^**^	−3.482	0.000	−0.372^**^	−3.707	0.000
Degradation	< – – –	Marriage length	−0.194^*^	−2.528	0.011	−0.278^**^	−2.802	0.005
Sexual Coercion	< – – –	Marriage length	−0.133	−1.734	0.083	−0.377^**^	−3.857	0.000
Property Damage	< – – –	Marriage length	−0.164^*^	−2.131	0.033	−0.196^*^	−1.997	0.046

** *p < 0.01*,

* *p < 0.05, two tailed*.

**Figure 3:**
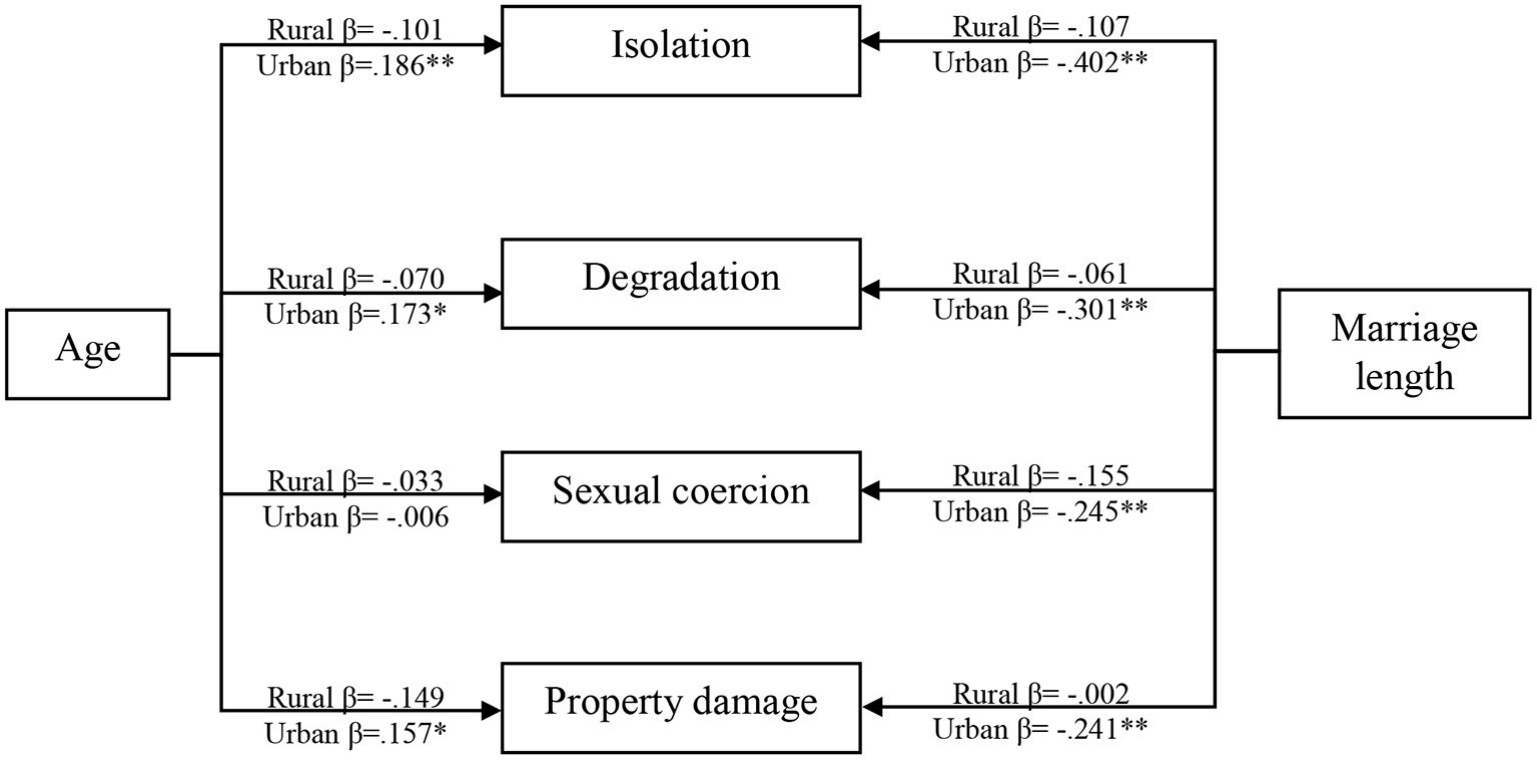
Multi-group modeling between rural and urban areas.

Multi-group modeling was again used to compare the effects of age and marriage length on the domains of emotional abuse across the two areas: rural (*n* = 198) and urban *(n* = 805). Compared to the fully constrained model (χ^2^ = 8597.092, df = 1,735), the unconstrained model better fitted the data (χ^2^ = 8535.012, df = 1,690). The chi-square difference between these two models was also significant, suggesting that the two groups (rural and urban) were not equal [Δχ^2^ = 62.08; Δdf = 45; *p* (d) = 0.046]. The results showed that no paths were significant in the rural group. However, all paths were significant in the urban group, except for the path between age and sexual coercion. Also, age had a significant positive impact on isolation, degradation, and property damage, suggesting that when age increases, emotional abuse in terms of isolation, degradation, and property damage also increases in urban areas. Finally, marriage length had a significant negative effect on sexual coercion, isolation, degradation, and property damage in urban areas, suggesting that emotional abuse decreases with marriage length. The results of multi-group modeling are presented in Table 10 and Figure 3.

**Table 10:** Multi-group modeling: interaction of area on associations of age and marriage length with emotional abuse victimization.

**Path relationship**			**Rural**			**Urban**		
			**Beta**	***T***	***P***	**Beta**	***T***	***P***
Isolation	< – – –	Age	−0.101	−0.752	0.452	0.186^**^	2.700	0.007
Degradation	< – – –	Age	−0.070	−0.524	0.600	0.173^*^	2.477	0.013
Sexual coercion	< – – –	Age	−0.033	−0.235	0.815	−0.006	−0.085	0.932
Property damage	< – – –	Age	−0.149	−1.097	0.273	0.157^*^	2.232	0.026
Isolation	< – – –	Marriage length	−0.107	−0.797	0.425	−0.402^**^	−5.688	0.000
Degradation	< – – –	Marriage length	−0.061	−0.455	0.649	−0.301^**^	−4.275	0.000
Sexual coercion	< – – –	Marriage length	−0.155	−1.107	0.268	−0.245^**^	−3.535	0.000
Property damage	< – – –	Marriage length	−0.002	−0.013	0.989	−0.241^**^	−3.428	0.000

** *p < 0.01*,

* *p < 0.05, two tailed*.

## Discussion

The emotional abuse of men in intimate relationships is a relatively unexplored area in the Arabic context, particularly in Jordanian society. Therefore, this study primarily aimed to fill this gap in the Arabic and Jordanian knowledge base through exploring the prevalence and forms of emotional abuse among Jordanian men in marital relationships. Furthermore, we investigated the roles of marriage length, marriage motivation, age, and residential area in emotional abuse.

In this study, married men reported experiencing different forms of emotional abuse. Among the various forms, isolation was the most prevalent, followed by degradation, property damage, and sexual coercion. These results are consistent with those of previous studies (Carney and Barner, 2012; Karakurt and Silver, 2013; Vinayak et al., 2015), which demonstrates that men experience emotional abuse by their intimate partner. Similarly, in Kasian and Painter's (1992) study, men reported experiencing more emotionally controlling behaviors, jealous behaviors, verbal abuse, and/or withdrawal from their partner than women. Isolation relates to psychological control (World Health Organization, 2013) that is used to facilitate control and power in pursuit of the following goals: weakening, intimidating, and devaluing victims; deriving pleasure from exercising power and control; gaining personal profit and personal gratification; and achieving psychological projection (Cory and McAndless-Davis, 2000; Lehmann et al., 2012). Abusers use various physical, social, and emotional tactics to exploit the vulnerabilities of victims and to render them extremely dependent on the abusers (Murphy, 2003; Braiker, 2004; Kantor, 2006). These tactics include economic abuse, emotional blackmail, verbal abuse, threats, inattention, nagging, and extortion (Braiker, 2004). Possessiveness and jealousy among partners are important motivations for isolating one's partner from social contact with friends and family (Murphy, 2003), which is achieved by controlling or limiting social and family interactions and activities, insisting on knowing where their partner is at all times, or controlling incoming information, including what they read. Other emotionally abusive behaviors include a partner complaining of unfaithfulness, getting angry, smearing and ignoring their partner, and forcing their partner to align with the partner abuser's beliefs and expectations (Murphy, 2003; Burgo, 2016).

Married men reported degradation as one of the most prevalent forms of emotional abuse within emotionally abusive relationships, with their wives divulging their secrets and personal information or telling other people that there is something wrong with them, ridiculing them, and comparing them unfavorably to other partners. Additionally, participants reported that their wives said things to hurt them out of spite, talked them into doing things that made them feel bad, and frequently questioned whether their love is true. Moreover, they reported that their wives physically threatened them during arguments. In the context of romantic relationships, Francis and Pearson (2019) ascertained that emotional abuse behaviors that involved personal degradation were perceived as the most serious/unacceptable, and both males and females tended to provide assertive responses for behaviors in the “personal degradation” domain, such as outright rejection, shaming their partner, putting down their partner's physical appearance and intelligence, refusing unreasonable requests, seeking help, asking the partner to stop their behavior, and stating personal rights. The emotional abuse of participants in the present study through property damage included several behaviors; most notably, wives threatened to break their husbands' valuable or prized belongings, and hurt people they care about.

Karakurt and Silver (2013) investigated the role of gender in emotional abuse within intimate relationships and found that women were more likely to experience property damage than men. Women's emotional abuse of their male partners may involve gender-based harassment involving behaviors that reinforce heteronormative gender roles, such as slurs, taunts, or name-calling related to a person's appearance or mannerisms, that do not fit stereotypical gender roles (Baker et al., 2015) and challenge men's ability to fulfill the male role (e.g., labeling a man as a woman) (Berdahl, 2007). Therefore, women may emotionally abuse men through manipulation, monitoring behaviors, and the adoption of different behavioral strategies in escalating arguments and violence through coercive verbal and non-verbal behaviors (insulting and swearing) (McHugh et al., 2013).

When we examined the assessment results of the sexual coercion subscale, we ascertained that despite its acceptable reliability, it yielded the lowest response scores. This might be due to differences in cultural norms and religion that regulate sexual activity. The Islamic religion and tribal culture play a role in shaping Jordanians' perceptions of sexuality. Conservative norms and the stereotype of masculinity in the socio-cultural Jordanian context have created cultural barriers that prevent men from expressing their emotions, perceiving themselves as victims of abuse, and reporting that they are victims of abuse by women, which is an unforgivable social stigma (Alsawalqa, 2021). Therefore, it is possible that participants were unable or unwilling to respond in a meaningful way to the Sexual Coercion subscale items (e.g., “My wife pressures me to have sex after an argument” and “My wife intentionally hurts me during sex”) and the “Degradation” subscale items (e.g., “My wife has told me that I am sexually unattractive,” “My wife threatens me physically during arguments,” “My wife intentionally does things to scare me,” and “My wife threatens me physically during arguments”), which would have played a role in the accuracy of the response scores. From another perspective, Follingstad (2007) argued that the concept of abuse could be misused, misunderstood, or manipulated due to several factors, such as interpretation, contextual, intention, common understanding, and multiplicity ideological, all of which influence the establishment of the concept of “psychological/emotional abuse” and render it ambiguous and intricate.

Therefore, recipients may not have an exact definition of emotional abuse or standard against which to judge the partner's behavior as abusive, especially if there is no intent to harm, and the importance of factors, such as abuse frequency, intensity, duration, and impact, for conceptualizing how they rate behavior as abusive. Follingstad (2007) indicated the different interpretations of psychological/emotional abuse measurement items, which sparked a debate about the possibility that respondents could be considered truly “abused” or even maltreated. For example, males' frequent agreement to the item “My partner is insensitive to my sexual needs” during their dating could be interpreted in several ways, that is, it could be that females might have been insensitive to reestablish a power differential, or males may have wished to engage in sex and labeled their partners “insensitive” when they were unwilling to cooperate. Without information to suggest that the dating partners' reactions went beyond “insensitivity,” spiraling into deliberate meanness or cruelty, we cannot label their experiences as “abusive.”

Our results revealed that emotional abuse decreased with age. Correspondingly, it has been reported that emotional abuse is more common among younger men (Karakurt and Silver, 2013). Women are renegotiating gender roles and expectations and are gaining access to similar resources as men. Therefore, they may engage in emotionally abusive behaviors in their intimate relationships as a tactic in their competitive struggle to gain control over scarce resources, while younger men may pay more attention to the rewards of the intimate relationship (e.g., access to sex), which could outweigh the conflict, possibly leading them to think that emotionally abusive behaviors do not warrant the label of “abuse”; accordingly, they may not perceive themselves as victims (Karakurt and Silver, 2013).

Our findings showed that emotional abuse decreased with longer marriage length, and marriage length had a significant negative effect on isolation, degradation, and property damage. This result can be interpreted in light of empirical evidence suggesting that marriage duration strengthens spouses' mutual recognition of each other's needs. Over the years, spouses would have lived diverse experiences together, which leads them to gain a better understanding of each other and their respective characters, learn how to accept things that are out of their control, and take daily action to deal with life's challenges. This may contribute to the reduced incidence of abuse (Alsawalqa, 2020). This result can also be linked to the aforementioned age-related results. In early (ages 20–40) and middle adulthood (ages 40–65), people wonder if they have made poor choices and what they should do with their lives, so they seek positive relationships to contribute to a state of well-being and may experiment with different aspects of their personality to seek out changes in their lives and learn how to cope with harmful behaviors. They find themselves and the meaning of their lives through work and family life, and their practical problem-solving skills increase.

Erik Erikson referred to this tendency as generativity vs. stagnation and intimacy vs. isolation (Boundless and Lumen Learning, 2021). Regarding marriage motivation, our results showed that emotional abuse was higher in love marriages than in traditional marriages and that individuals in both traditional and love marriage experienced higher emotional abuse in rural areas than in urban areas. This result, which was not surprising, may be due to the prevalence of the participatory knowledge of the concept of love in urban areas, which is knowing by becoming. It comes with a certain degree of imitation and internalization, so there are many stories about what love is or how it should be. Away from the scientific understanding of what they feel, individuals form their concept of love based on models of love relationships they observe around them in their parents and relatives, from television and movies, from conversations with other people about their relationships, fairy tales, and personal experiences. People in urban areas may have greater access to movies, and other media sources that depict love in a non-abusive way compared to those in rural areas. People in rural areas may be less influenced by media and rely more on observations of parents and other family members. This has led to many disagreements about love's precise meaning (Sternberg, 2021). Scientifically, love as a complex concept can be understood and analyzed. Turner and Stets (2005) discussed the argumentation over the biological and cultural basis of emotions and defended the idea that the theoretical study of emotions is the key to understanding rationality. They explained that emotions are socially constructed, meaning that cultural ideologies, beliefs, and norms define what emotions are to be experienced and how these culturally defined emotions are to be expressed (p. 2). Thus, emotions emerge from intimate social situations, and through the socialization process, individuals learn an emotion vocabulary that enables them to name internal sensations associated with objects, events, and relations that they encounter and learn the appropriate emotions and how to use them in different types of relationships. For example, jealousy signals the intrusion of another into a valued relationship. We learn to apply the label “love” to situations where autonomic symptoms such as the flow of adrenaline and an increased heart rate occur in the presence of another who we perceive as attractive. Vocabularies of emotions may differ among cultures, and people cannot distinguish between “liking” and “loving” (p. 3).

The definition of love depends on the cultural context, which plays a main role in influencing the expectations we have of what love should be like through sociodemographic differences such as gender, race, economic status, religion, education, and ethnicity (Ridgeway et al., 2009). These sociodemographic factors may account for different prevalence rates of emotional abuse between rural and urban areas in this study. The items of the emotional abuse questionnaire employed in this study that received the highest ratings from men who married out of love led us to predict how abusive women express their love for their husbands, which appears to be through acts of jealousy, restricting men's movements, and questioning their love. Jealous behaviors seem to be common among Jordanian women; some women interpret jealousy as a sign of love (Croucher et al., 2012). According to Turner and Stets (2005), primary emotions can lead to secondary emotions (e.g., joy and acceptance leads to love and friendliness). By that analogy, love may lead to jealousy, which, in turn, may lead to emotional abuse (Puente and Cohen, 2003). Culturally, jealousy may be used to justify violence or aggression toward partners. Whether the jealousy felt is normal or morbid is often not crucial; an individual's other characteristics, such as impulsiveness and insensitivity, are the main factors that lead to violence (Mullen, 1995). Filho et al. (2019) suggested that jealousy combined with anxiety may contribute to IPV. Love can cause people to become victims of emotional abuse; if they love the wrong person, love will be the source of hurt or suffering, heartbreak, and regret (Milligan, 2011), or they might find it difficult to differentiate between their emotions or distinguish between the types of love, including unconditional, sexual, and mature love. Sexual love is a basic human instinct (Murphy, 2002; Singer, 2009), while mature love occurs when spouses are together because they want to be together and not just because they feel an irrational desire or need to be with one another (Stritof, 2020). They may also easily fall in love when they are emotionally aroused during challenging times.

Regarding gender differences in emotional experience and expressivity, Deng et al. (2016) found that women reported higher arousal levels when watching videos that induced anger, sadness, amusement, and pleasure. Men often have more intense emotional experiences, whereas women have higher emotional expressivity, particularly for negative emotions. Husbands and wives use relationally aggressive tactics when dealing with conflicts in their marriage, such as social sabotage and love withdrawal, and wives are more likely to be relationally aggressive than husbands. Additionally, relational aggression is associated with lower levels of marital quality and greater marital instability in spouses (Carroll et al., 2010). Also, marital satisfaction is associated with empathic accuracy, which refers to one's ability to accurately infer and perceive their partner's thoughts, feelings, and other internal mental states (Sened et al., 2017). Lower empathic accuracy for a partner's negative emotions may adversely affect the perceiver's marital satisfaction (Sened et al., 2017). In a sample of couples with a history of partner aggression, Cohen et al. (2015) found that lower empathic accuracy in women was associated with greater physical and psychological aggression in both men and women. Moreover, women's inaccuracy in reading their partner's hostility was linked to women's greater psychological aggression toward men, while men's inaccuracy in reading their partner's hostility was linked to women's (not men's) greater physical and psychological aggression. Future studies should adopt additional indicators, such as emotional neglect and empathic accuracy, to identify the developmental course of intimate relationships that lead to emotional abuse. Little is known about the associations between marriage motivation and emotional abuse in different cultures, which may serve as new paths to broader outcomes. Furthermore, especially in Arab cultures, religion may be essential to a better understanding of what is normative in identifying the concept of love and marriage and its motivation; religion may play a moderating role in the path to emotional abuse.

## Strengths and Limitations

This study investigates the moderating roles of marriage length and motivation on emotional abuse within marital relationships. One of its strengths is that it was conducted with a large and diverse sample of men of varied demographic backgrounds, as one of the first studies to address female-perpetrated emotional abuse. Within the social-cultural context, most view women's violence toward intimate partners ambivalently. Domestic abuse is often treated as a female-centric issue and this study contributes toward disproving this notion. Nevertheless, the present study also has some limitations. First, including women in the study sample could have enhanced the results and clarified details on the roles of marriage length and motivation, and on examining the interplay between spousal interactions through analysis such as the actor–partner interdependence model, which would increase our understanding of the rationale behind the emotional abuse perpetrated by both the partners. Second, the results should be considered in light of the limitations of including a sample of married men. Also, including additional variables, in conjunction with kinship between spouses, wives' age, educational level, and employment status could provide value in terms of understanding the differences in the paths of emotional abuse within marital relationships. Finally, after an extensive review of emotional abuse measures in the field of IPV, the present study's scale was the one that best fit the objectives of the study and the context under examination, despite its acceptable reliability. The sexual coercion subscale negatively reflected on the total score of emotional abuse. This was due to cultural norm and religious differences that regulate sexual activity in the socio-cultural Jordanian context, creating cultural barriers that led to self-report bias. Moreover, emotional abuse scales need to include items regarding children; due to its inclusion in previous studies, it is as an important tactic in the emotional abuse of men by women and merits further research. The biggest question that should be mentioned in terms of future research is what effect does this emotional abuse have on men's marital satisfaction? It would also be interesting for future research to include characteristics of the men who are on the receiving end of emotional abuse tactics; for example, assessing personality (such as The Big Five) or attachment style or even The Dark Triad personality traits may suggest how men's own behavior or personality tendencies might intensify or attenuate women's abusiveness toward their husbands.

## Conclusions

For years, people believed that only women were subjected to emotional abuse in their marriages or relationships. It is agreed that there are many more women and children abused worldwide than men. However, one cannot deny the fact that men also suffer emotional abuse by women, and we must recognize this issue, raise awareness of it, and combat it. Thus, this study investigated the experience of emotional abuse of 1,003 men from Amman city, Jordan, who were selected randomly using a probability sampling strategy. They reported experiencing emotional abuse in their marital relationships by their wives through isolation and degradation, including insults, verbal attacks, financial coercion, threatening behavior, labeling, and coercive control. Further study is required to explore the nature of emotional abuse forms and their underlying reasons among married men in the Arabic cultural context, as differences in sociodemographic characteristics such as kinship between spouses, wives' age, educational level, and employment status could affect the identification and understanding of emotional abuse and contribute to developing an intellectual framework capable of finding solutions for abusive marital relations in the Jordanian context.

## Data Availability Statement

The original contributions presented in the study are included in the article/supplementary material, further inquiries can be directed to the corresponding author/s.

## Ethics Statement

The studies involving human participants were reviewed and approved by the Institutional Review Board (IRB) of the University of Jordan, the Hashemite Kingdom of Jordan (reference number IRB/19/2021/86). The ethics committee waived the requirement of written informed consent for participation.

## Author Contributions

RA conceived and designed the experiments, performed the experiments, contributed reagents, materials, analysis tools or data, and wrote the paper. YS contributed reagents, materials, analysis tools, or data. MA contributed materials, analysis tools, or data, and revising the work. All authors contributed to the article and approved the submitted version.

## Conflict of Interest

The authors declare that the research was conducted in the absence of any commercial or financial relationships that could be construed as a potential conflict of interest.

## Publisher's Note

All claims expressed in this article are solely those of the authors and do not necessarily represent those of their affiliated organizations, or those of the publisher, the editors and the reviewers. Any product that may be evaluated in this article, or claim that may be made by its manufacturer, is not guaranteed or endorsed by the publisher.
